# Preparation and Characterization of WS_2_@SiO_2_ and WS_2_@PANI Core-Shell Nanocomposites

**DOI:** 10.3390/nano8030156

**Published:** 2018-03-10

**Authors:** Hagit Sade, Jean-Paul Lellouche

**Affiliations:** Institute of Nanotechnology and Advanced Materials & Department of Chemistry, Faculty of Exact Sciences, Bar-Ilan University, Ramat Gan 5290002, Israel; Hagit.Sade@biu.ac.il

**Keywords:** WS_2_, functionalization, core-shell, polyaniline, silica

## Abstract

Two tungsten disulfide (WS_2_)-based core-shell nanocomposites were fabricated using readily available reagents and simple procedures. The surface was pre-treated with a surfactant couple in a layer-by-layer approach, enabling good dispersion of the WS_2_ nanostructures in aqueous media and providing a template for the polymerization of a silica (SiO_2_) shell. After a Stöber-like reaction, a conformal silica coating was achieved. Inspired by the resulting nanocomposite, a second one was prepared by reacting the surfactant-modified WS_2_ nanostructures with aniline and an oxidizing agent in an aqueous medium. Here too, a conformal coating of polyaniline (PANI) was obtained, giving a WS_2_@PANI nanocomposite. Both nanocomposites were analyzed by electron microscopy, energy dispersive X-ray spectroscopy (EDS) and FTIR, verifying the core-shell structure and the character of shells. The silica shell was amorphous and mesoporous and the surface area of the composite increases with shell thickness. Polyaniline shells slightly differ in their morphologies dependent on the acid used in the polymerization process and are amorphous like the silica shell. Electron paramagnetic resonance (EPR) spectroscopy of the WS_2_@PANI nanocomposite showed variation between bulk PANI and the PANI shell. These two nanocomposites have great potential to expand the use of transition metals dichalcogenides (TMDCs) for new applications in different fields.

## 1. Introduction

Nanostructures of tungsten disulfide (WS_2_) were discovered 25 years ago [1] and have been continuously studied ever since. WS_2_ inorganic nanotubes (INTs) and fullerene-like nanoparticles (IFs) are closed cylindrical or polyhedral layered structures. WS_2_ is a part of a chemical group of compounds called transition metal dichalcogenides (TMDC), which are sulfides, selenides and tellurides of group 5 and 6 transition metals. In the same manner that carbon nanotubes (CNTs) are composed of folded graphene sheets, WS_2_ nanostructure are composed of folded triple-layer S-W-S sheets [2]. The atoms within each triple layer are covalently bonded. Between the triple layers, however, there are weak van der Waals interactions, meaning that these nanostructures are of an anisotropic nature. Due to this anisotropy, WS_2_ nanostructures (especially IFs) are often added to liquid lubricants for reduction of friction and wear [3,4,5,6,7]. The weak forces between the triple layers facilitate sliding and exfoliation of the layers under rubbing pressure.

Inorganic nanostructures are an excellent alternative to CNTs for mechanical reinforcement. WS_2_ nanostructures have high strength moduli and a good ability for shock absorbing [7]. These mechanical properties allow their use for reinforcement of different polymeric matrices [8,9,10].

In addition to the tribological and mechanical advantages of WS_2_ nanostructures, they have low cytotoxicity and high biodegradability [11,12,13], unlike CNTs. This adds the possibility of using WS_2_ nanostructures in biological and medical applications.

Surface functionalization of nanostructures in general and of TMDC nanostructures in particular, is important for two main reasons: First, the functionalized nanostructures will have a better ability of incorporation in different media and matrices. Second, functionalization can add beneficial attributes to the nanostructure, resulting in a larger number of possible applications of the nanocomposite compared to the bare nanostructure. For TMDC nanostructures specifically, the following examples from the literature will demonstrate the benefit in surface functionalization.

Tremel, Tahir and their co-workers have been researching in this field of TMDC functionalization for over a decade now. They developed a general technique for the functionalization of TMDC nanostructures that employs a nitrilotriacetic acid (NTA) ligand to immobilize functional molecules on the outer sulfur layer [14]. Because of the difficulty to anchor organic ligands to the relatively inert sulfur surface, a nickel cation, which has a high sulfur affinity and an octahedral coordination is used. Chelation of the tetradentate NTA with the cation leaves the rest of the nickel coordination sphere free for docking to the sulfur layer. A variety of chemical moieties can be attached to NTA. In other words, Ni-NTA has high affinity to TMDC walls from one end and versatile functionality from the other end. Variations of this technique were used for dispersion of MoS_2_ IFs in water [14], for attachment of titania nanoparticle to WS_2_ INTs [15], for binding of the biocatalysts protein silicatein to WS_2_ INTs [16] and for conjugation of protoporphyrin to ReS_2_ IFs [17]. By a similar metal-ligand principal, Fe-terpyridine was used on ReS_2_ IFs to increase their dispersibility in different solvents [18]. Another strategy developed in the Tremel group utilizes the HSAB (Hard Soft Acid Base) principle by Pearson. According to this principle, a hard Lewis acid has a tendency to bind with a hard Lewis base and vice versa [19]. The “soft” sulfur surface layer has then a tendency to bind with other nanoparticles containing soft transition metal cations. This strategy was used for reversible functionalization of WS_2_ nanotubes and MoS_2_ IFs with MnO, Fe_2_O_3_, Fe_3_O_4_, ZnO, gold and platinum nanoparticles [20,21,22]. Shahar et al. used surface defects to functionalize WS_2_ IFs with alkyl–silane molecules for better dispersion in oil-based suspensions [23] and to attach gold nanoparticle to WS_2_ INTs [24]. Polyakov et al. decorated MoS_2_ and WS_2_ nanostructures with gold nanoparticles by adding a suspension of the nanostructure into boiling aqueous solutions of HAuCl_4_ [25]. Next, two more optional types of functional shells for TMDC nanostructures will be discussed—silica and polyaniline.

Silica coating and amorphous silica coating in particular, is one of the best, most studied and most versatile types of functional shells. A partial list of its advantages includes chemical and thermal stability, biocompatibility, reduced toxicity and optical transparency. A silica coating is relatively easy and inexpensive to prepare. Additionally, it improves dispersibility in a wide range of media, especially aqueous. It also provides the option for further surface modification of the nanocomposite through the use of functional silicates and attachment of different molecules, such as dyes, to the coating. The literature contains a large number of applications for silica-coated nanomaterials, in a variety of fields. Presented here are a few examples. Silica coating enhanced emission of NdF_3_ nanoparticles [26], NaYF_4_:Yb^3+^, Er^3+^ nanoparticles [27] and CuO nanorods [28]. Dispersibility and biocompatibility of luminescent YF_3_:Tb^3+^ nanoparticles [29], LaVO4:Eu^3+^ nanoparticles [30] and LaPO_4_:Eu@ LaPO_4_ nanorods [31] were improved by silica coating, increasing their potential uses in biomedical science. Silica coating prevented aggregation of magnesium ferrite [32] and cobalt ferrite [33] nanoparticles, while maintaining their magnetic properties. Fe_3_O_4_ nanoparticles with a siliceous shell were used for removing heavy metal ions from water [34,35]. A gas-phase silica encapsulation technology was applied to reduce the toxicity of fumes generated by flame [36] or welding [37]. Ultrafine diamonds, used for ultra-precision grinding, were coated with silica to improve their oxidation resistance [38]. Silica coating reduced the toxicity of CeO_2_ [39,40] and ZnO [41] nanoparticles. Due to its good specific charge capacity, silicon-based electrodes coated with silica can be incorporated in next-generation lithium-ion batteries [42]. 

Polyaniline (PANI) belongs to a group of conducting organic polymers (COPs), which have been extensively studied and utilized in the last four decades [43]. This group also includes polypyrrole (PPy) [44,45], polycarbazole (PCBz) [46,47], polythiopene (PTh) [48,49], poly [3,4-ethylenedioxythiophene] (PEDOT) [48,50] and others. These polymers (also referred to as synthetic metals) have superior electrical properties and mechanical strength; they are also flexible, environmentally stable and thermally stable. 

PANI has been the most attractive and by far, the most studied polymer out of the COPs. In addition to the characteristics described above, PANI is easy to synthesize, inexpensive to produce, structurally diverse, light-weight and corrosion-resistant [51,52,53]. PANI is used for catalysis [54,55], fuel cells [56,57], solar cells [58], supercapacitors [59], sensors [60], corrosion protection [61], printing inks [62], rechargeable batteries [63] and the list goes on. PANI has been incorporated in nanocomposites with graphene [64,65], carbon nanotubes [66,67], metals [68,69,70,71], metal oxides [72,73,74], metal sulfides [75,76,77] and many other materials. Focusing on PANI incorporation with TMDCs, MoS_2_-PANI nanocomposites have been prepared by in-situ procedures [78,79]. WS_2_ nanosheets [80] and nanotubes [81] have been incorporated into a PANI matrix.

In this paper, we report the preparation and characterization of two new core-shell nanocomposites—WS_2_@SiO_2_ and WS_2_@PANI. The nanocomposites were prepared using WS_2_ INTs and IFs that underwent a preliminary layer-by-layer treatment with a surfactant pair, which enabled further polymerizations in polar media. The nanocomposites were analyzed by various methods to verify the formations of the shells and learn about their characteristics. The preparation protocols we present here are not only simple, making use of readily available reagents, instrumentation and techniques and allow the formation of two highly functional polymeric shells but also serve as a synthetic platform for further developments in TMDC functionalization. The pre-treatment alone opens the option for growth of other hydrophilic polymer shells on TMDC nanostructures. The growth of the shells does not rely on surface defects. As a result, they are conformal and have good coverage on the WS_2_ nanostructures surface. Shell properties, such as thickness and morphology, may be controlled by changing polymerization parameters. Some modifications can be applied in the preparation procedures to produce more TMDC-based core-shell nanocomposites: The core TMDC may vary; substituted silica and PANI monomers can be used in the polymerization process; functional materials/nanoparticles can be encapsulated into the shell. Improved properties and additional uses are among the advantages that core-shell nanocomposites have over the core nanostructure, both in general and for the nanocomposites presented here. For example, fibers and coatings reinforced by TMDC nanostructures will have the added values of antibacterial effect from silica and of electrical conductivity from PANI. This opens possible applications for the nanocomposites in the biomedical field, electrochemistry, textile, plastics and more.

For these reasons, we believe that this work is beneficial. We are hopeful that these nanocomposites will inspire other first- and second-level surface functionalizations of WS_2_ and other TMDC nanoparticles and expand their existing uses.

## 2. Experimental Section

Silica coating is usually produced by polymerization of TEOS using a Stöber-like method, in reverse microemulsion [30,82] or sol-gel [26,29,31,32,33,83] setups.

In some cases, TEOS can be polymerized directly on the surface of the core material [34,35]. In other cases, a mediation of a surfactant is needed [27,38,84,85,86].

An attempt to polymerize TEOS directly on WS_2_ nanostructures did not succeed. TEOS polymerization is done in a polar medium. While such a medium is excellent for the polymerization of TEOS to silica, it is far from ideal for proper dispersion of WS_2_ nanostructures. The nanostructures rapidly aggregate and sink and the formation of silica nanoparticles separately from the WS_2_ nanostructures is kinetically favored. To increase the affinity of WS_2_ towards TEOS, we tried using a protocol previously used to functionalize carbon nanotubes with a mesoporous silica shell [87]. The idea is a layer-by-layer (LbL) coating method wherein two surfactants are used alternately: PSS (poly [sodium p-styrenesulfonate]) and CTAB (cetryltrimethyl ammonium bromide). PSS has some affinity to the WS_2_ nanostructure walls through hydrophobic interactions. Although one cycle of pre-treatment with the surfactant couple is insufficient to achieve good affinity towards TEOS, five sequential cycles provide a much better coverage and an ionic shell is formed on the nanoparticle. This shell allows increased dispersibility in polar media and serves as a template for silica polymerization.

Polymerization of aniline takes place in an aqueous medium, in which, as mentioned, WS_2_ nanostructures are not well-dispersed. After the success of the silica coating procedure, we were able to prepare a WS_2_-PANI core-shell nanocomposite using the surfactant-modifies nanoparticles.

**Scheme 1:**
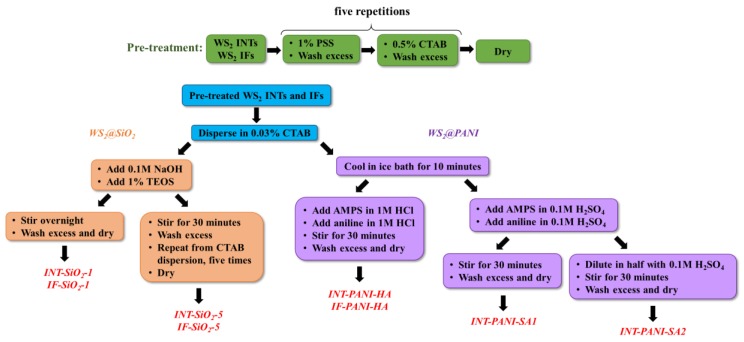
A summary of the experimental procedure for preparation of WS_2_@SiO_2_ and WS_2_@PANI nanocomposites samples.

Scheme 1 below summarizes the experimental pathway and full details are given in the following sections.

### 2.1. Layer-by-Layer Surface Modification of WS_2_ Nanostructures

An aqueous 1% (*wt/wt*) PSS solution was prepared by mixing 10 g of PSS (Lot # BCBR9107V, average Mw ~70,000, Sigma-Aldrich, St. Louis, MO, USA), with 990 g of water. The water used for all preparations in this section is ultrapure, purified by a Merck Milli-Q^®^ system (Merck & Co., Kenilworth, NJ, USA).

An aqueous 0.5% (*wt/wt*) CTAB solution was prepared by mixing 5 g of CTAB (Lot # 054K0083, Sigma-Aldrich, St. Louis, MO, USA) with 995 g of water. Both solutions were sonicated and well-shaken to homogeneity. 

5 g of WS_2_ INTs or IFs (Lot numbers TWPO-MA018 and FWPO-HC019, respectively; NanoMaterials Ltd., Yavne, Israel) were equally divided between four 50 mL conical plastic tubes. 30 mL of 1% PSS solution was added to each tube. To disperse the nanoparticles in the PSS solution, the tubes were sonicated for 20 min in a sonication bath, followed by 30 s of vortex. After dispersion, the tubes were centrifuged for 20 min (7500 RPM, 5 °C) and the liquid was decanted. Then the nanoparticles were washed (same conditions) with 30 mL water, to remove residual PSS. Next, the nanoparticles were dispersed in 30 mL of 0.5% CTAB solution, centrifugation and washing with 30 mL water. This cycle of PSS, water, CTAB and water, was repeated four more times to a total of five cycles. Finally, the contents were washed with 30 mL of ethanol absolute (ACS reagent, Carlo Erba, Milan, Italy) and dried overnight in a vacuum oven at 60 °C.

### 2.2. Preparation of WS_2_@SiO_2_ Nanocomposites

50 mg of nanoparticles after layer-by-layer coating were weighed into a 20 mL vial and dispersed in 10 mL of 0.03% (*wt/wt*) aqueous CTAB solution (diluted from the 0.5% solution), by 10 min of sonication. A 0.1 M NaOH aqueous solution was prepared by mixing 1 mL of 1 N NaOH standard solution (Lot #: 7576–3700, Daejung, Busan, South Korea) with 9 mL water. From the diluted NaOH solution, 100 µL were then added to the vials while stirring on a plate.

A solution of 100 µL TEOS (puriss, ≥99.0%, Lot #: 1332815, Fluka, St. Louis, MO, USA) in 10 mL ethanol absolute was added to the vial dropwise, using a glass pipette. The vials were left to stir overnight at room temperature (750 RPM). 

The next day, the contents were washed with five portions of 30 mL water, followed by 3 portions of 30 mL ethanol absolute (2500 RPM, RT, 15 min). Low speed was chosen to separate between the nanotubes and any silica that might have polymerized in the solution. The contents were then dried overnight in a vacuum oven at 60 °C. These samples are marked INT-SiO_2_-1P and IF-SiO_2_-1P.

Alternately, we tried using 5 portions of 10 mL ethanolic TEOS solution, in intervals of 30 min. After each interval, the content was centrifuged (2500 RPM, RT, 15 min) and re-dispersed in 10 mL 0.03% CTAB solution. The final washing and drying steps remained the same. This procedure resulted in a slightly thicker and denser silica coating. These samples are marked INT-SiO_2_-5P and IF-SiO_2_-5P.

### 2.3. Preparation of WS_2_@PANI Nanocomposites

Aniline polymerization was done in diluted acidic media: hydrochloric acid (HCl) or sulfuric acid (H_2_SO_4_), using ammonium persulfate (AMPS) as an oxidizing agent.

A 1M HCl solution was prepared by adding 32% HCl solution (Lot #: 4710-4100, Daejung, Busan, South Korea) to water, filling to 100 mL and mixing. A 0.1M H_2_SO_4_ solution was prepared by adding 98% H_2_SO_4_ solution (Lot #: 7683–4100, Daejung, Busan, South Korea) to water, filling to 100 mL and mixing. Acidic 0.2M aniline solutions were prepared by adding 91.21 µL of aniline (ACS reagent, 99+%, Lot #: R30A727, Alfa Aeser, Haverhill, MA, USA) to each of the diluted acidic solution mentioned above, filling to 5 mL and mixing.

Acidic 0.25M AMPS solutions were prepared by adding 285.25 mg of AMPS (ACS reagent, 98+%, Lot #: 09406TQ, Sigma-Aldrich, St. Louis, MO, USA) to each of the diluted acidic solution mentioned above, filling to 5 mL and mixing. The aniline and AMPS solutions were freshly prepared for the experiments.

For the coating procedure, 50 mg of nanoparticles after layer-by-layer coating were weighed into a 20 mL vial and dispersed in 10 mL of 0.03% CTAB solution by 10 min of sonication. The vial was then cooled in an ice bath for 10 min. Next, 5 mL of acidic AMPS solution were added, followed by 5 mL of acidic aniline solution. The vials were left to stir (650 RPM) for 30 min at room temperature. The contents were then washed with 30 mL portions of water until washing liquid faded from blue to almost colorless, followed by 2 portions of 30 mL ethanol absolute (1500 RPM, RT, 5 min). The contents were then dried overnight in a vacuum oven at 60 °C. We also tried an experimental variation on the INTs using diluted solutions: 2.5 mL of each acidic solution were used (AMPS and aniline) and 5 mL of the acid solution was added. The rest of the procedure was the same. For HCl solutions, we obtained no coating. For H_2_SO_4_ solution, we obtained a slightly thinner coating compared to the original quantities. The samples were marked as follows: INT-PANI-HA and IF-PANI-HA for the samples prepared in hydrochloric acid medium; INT-PANI-SA1 and INT-PANI-SA2 for the INT samples prepared in sulfuric acid medium (diluted and non-diluted, respectively).

### 2.4. Characterizations

*Transmission electron microscopy (TEM)* images were acquired by a Tecnai Spirit Bio-Twin microscope (FEI, Hillsboro, OR, USA) equipped with a 1 × 1k CCD camera (Gatan, Pleasanton, CA, USA). Samples for TEM analysis were dispersed in ethanol. A drop of the dispersion was placed on a formvar/carbon film on 400-mesh copper TEM grid (FCF400-Cu, Electron Microscopy Sciences, Hatfield, PA, USA) and dried at room temperature for 2–3 h.

*High-resolution scanning electron microscopy (HRSEM)* images and elemental line scans were acquired using Magellan 400L high resolution scanning electron microscope (FEI) equipped with an EDS detector. Samples for HRSEM were prepared by placing a few drops of a 0.5 mg/mL ethanolic solution of the dried sample on a square piece of a clean silicon wafer, then coating the sample with 3 nm of sputtered iridium. The silica-coated sample analyzed for elemental line scanning was prepared on a copper grid.

*Thermogravimetric analysis (TGA)* was performed using a TGA/DSC1 analyzer (Mettler-Toledo, Greifensee, Switzerland). The thermograms were recorded in a nitrogen (50 mL/min) environment at a heating rate of 10 °C min^−1^ over the temperature range 25–800 °C. The results were processed using STARe evaluation software (Mettler-Toledo, Greifensee, Switzerland).

*ATR-FTIR* spectra were obtained on a Nicolet iS5 FT-IR spectrometer (Thermo Scientific, Waltham, MA, USA) equipped with an iD5 ATR accessory featuring a laminated diamond crystal. Samples were analyzed as-is. Data processing was performed using OMNIC 9 spectra software (Thermo Scientific, Waltham, MA, USA).

*Zeta potential measurements* were performed using a Zetasizer Nano-ZS device (Malvern Instruments Ltd., Worcestershire, UK). Samples for zeta potential measurements were dispersed in deionized water (~0.1 mg/mL) and sonicated for five minutes in an ultrasonic bath.

*BET (Brunauer–Emmett–Teller) specific surface area (SSA)* was determined from nitrogen (N_2_) adsorption/desorption isotherms measured at liquid nitrogen temperature (−196 °C) using a Nova 3200e^®^ analyzer (Quantachrome instruments, Boynton Beach, FL, USA). The samples were degassed for two hours at 120 °C prior to the analysis. Specific surface area, pore volume and average pore size were determined using the Barrett−Joyner−Halenda (BJH) model. The data were processed using NovaWin™ software (Quantachrome instruments, Boynton Beach, FL, USA).

*X-band EPR* spectra were recorded at room using an X-band Elexsys E500 EPR spectrometer (Bruker, Karlsruhe, Germany) equipped with an integrated frequency counter. The powdered samples were inserted into narrow quartz tubes (2 mm OD 1 mm ID, Wilmad LabGlass, Vineland, NJ, USA) and placed within standard rectangular Bruker EPR cavity (ER 4119 HS). The EPR device was operated at a microwave frequency of ∼9.88 GHz. Spectra were recorded using a microwave power of 20 mW and gain 120 across a sweep width of 100 G with modulation amplitude of 2 G. Fitting was obtained by using the curve fitting function of the Xepr acquisition software (Bruker, Billerica, MA, USA), for suitable line shapes. 

## 3. Results and Discussion

**Figure 1:**
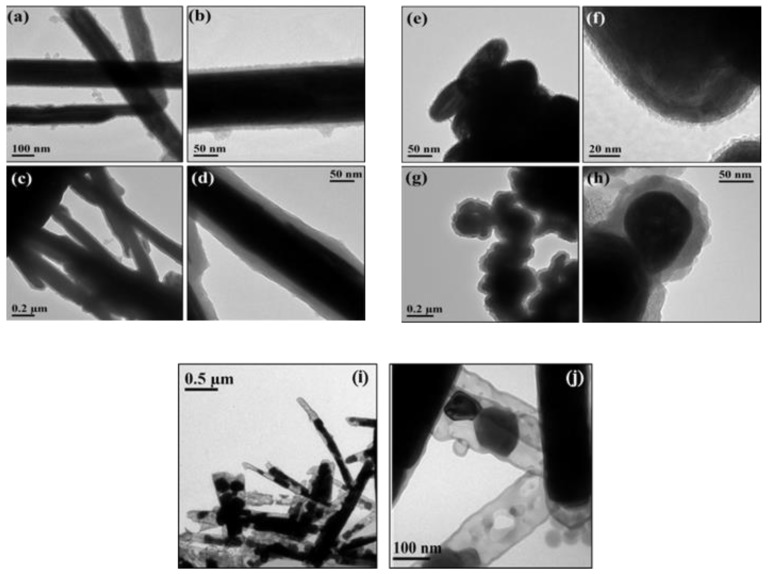
Transmission Electron Microscopy (TEM) images of WS_2_@SiO_2_ nanocomposites: INT-SiO_2_-1P (**a**,**b**); INT-SiO_2_-5P (**c**,**d**); IF-SiO_2_-1P (**e**,**f**); IF-SiO_2_-5P (**g**,**h**) and INT-SiO_2_-1P after heating at 600 °C for 3 h under air (**i**,**j**).

TEM images of WS_2_@SiO_2_ nanocomposites (Figure 1a–h) show that a conformal silica shell was formed around the WS_2_ INTs and IFs. For the procedure with the smaller amount of TEOS (a,b,e,f), the shell is a few nanometers thick and has a flake-like structure. For the procedure with the larger amount of TEOS (c,d,g,h), the shell is tens of nanometers thick and appears to be denser and smoother. The silica coating is amorphous, as no electron diffraction pattern was obtained from it. To verify the presence of the silica shell, we heated sample INT-SiO_2_-1P at 600 °C for three hours, under air atmosphere. For comparison, carbon nanotubes are burnt under these conditions, leaving behind hollow SiO_2_ nanotubes [75]. Under the same conditions, WS_2_ is merely oxidized to WO_3_ and the silica stays intact. Hence, we expected at least some change in appearance of the heated sample. TEM images of sample INT-SiO_2_-1P after heating (Figure 1i,j) show that indeed, the core material has partially collapsed and hollow parts of the silica shells are clearly observed. There are holes in different areas of the shell, which may be a result of particle coating and/or are caused during the WS_2_ collapse.

**Figure 2:**
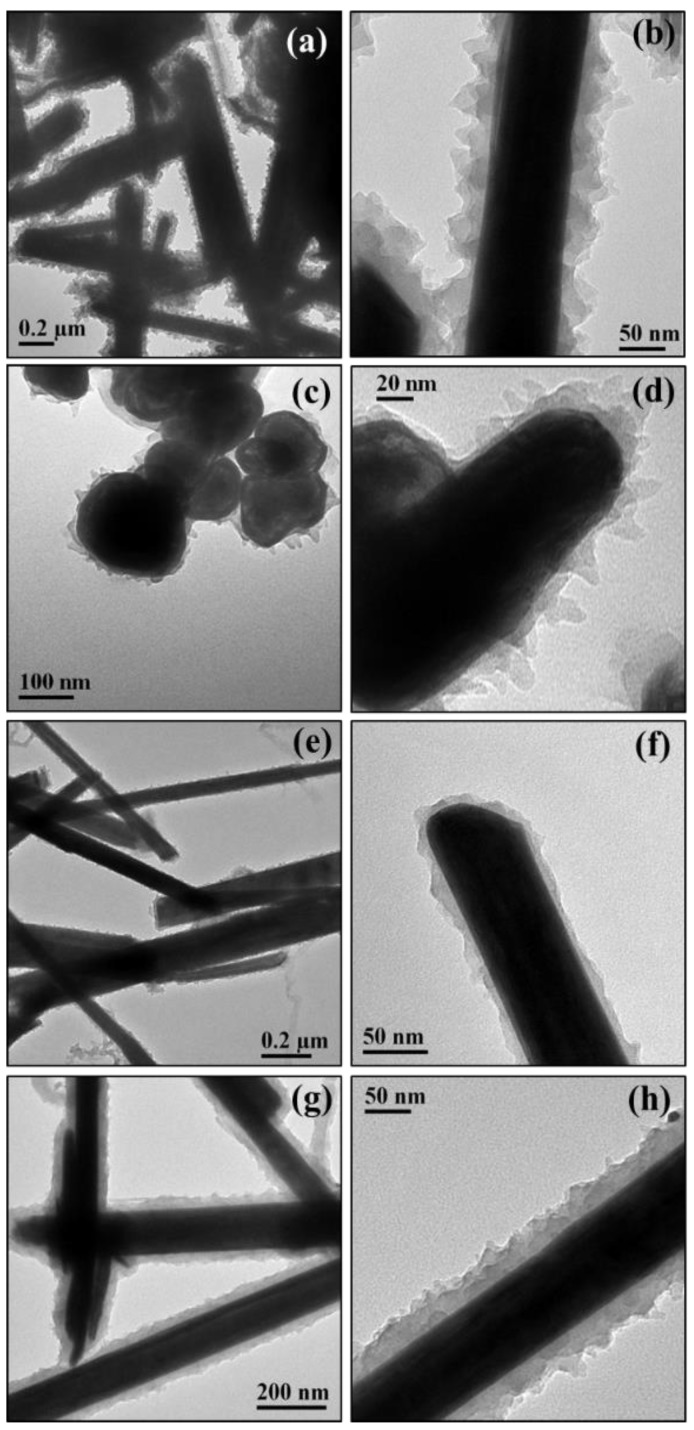
TEM images of WS_2_@SiO_2_ nanocomposites: INT-PANI-HA (**a**,**b**); IF-PANI-HA (**c**,**d**); INT-PANI-SA1 (**e**,**f**); INT-PANI-SA2 (**g**,**h**).

TEM images of WS_2_@PANI nanocomposites (Figure 2) show a uniform, conformal shell formed around the core WS_2_ INTs and IFs. Similar to the silica shell, the PANI shell seems to coat individual nanoparticles. For the procedure done with hydrochloric acid (a–d), PANI seems to grow in a nano-leaf, or a nano-petal type of structure, where the shape of the “petal” resembles a triangle with its base attached to the core nanostructure and its edge pointing outside. As a result, the “petals” are relatively loosely packed. For the procedure done with sulfuric acid (e–h), the coating seems to be composed of more densely-packed nanosheets. In other publications [88,89], the model of directional growth was used to explain different morphologies of aniline polymers. According to the model, different nano- and micro-structures are formed from aniline oligomers depending on the balance of attractive forces between the oligomer molecules. These forces can be affected by the medium surrounding the oligomers. In our case, it might be possible that the denser packaging is a result of π-π stacking attractive forces, meaning the aniline molecules are oriented in a way that the benzene rings are facing each other. The looser packaging can result from an orientation where the amine groups of the aniline molecules repulse each other. For both preparation procedures, the obtained coating produces no electron diffraction, meaning it is amorphous.

**Figure 3:**
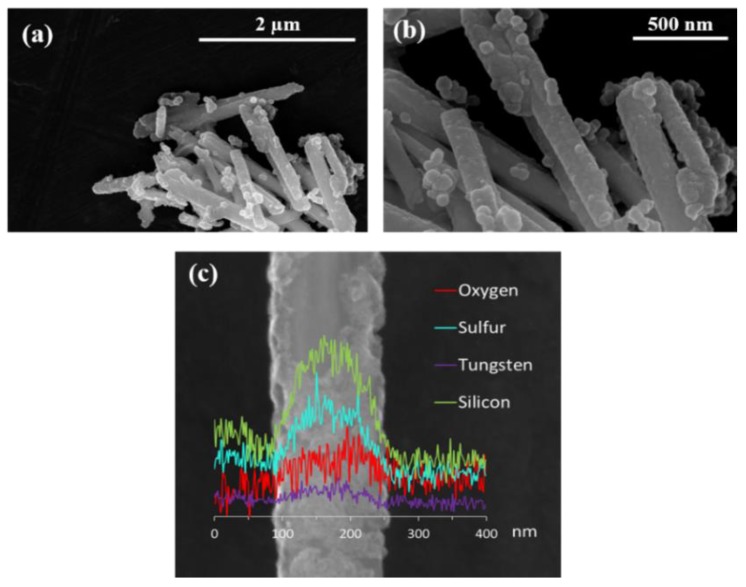
*High-resolution scanning electron microscopy* (HRSEM) images (**a**,**b**) and energy dispersive X-ray spectroscopy (EDS) line scan (**c**) of sample INT-SiO_2_-1P.

HRSEM images and EDS line scan of sample INT-SiO_2_-1P (Figure 3) confirm the presence of a conformal shell. The flaky and somewhat incomplete structure of the thin shell is more clearly visible from these images. The shell seems to be coating individual, rather than bundled, nanotubes. Although the shell is only a few nanometers thick, the EDS line scan (c) shows very clear signals of silicon and oxygen going hand in hand with the tungsten and sulfur signals of the nanotube.

**Figure 4:**
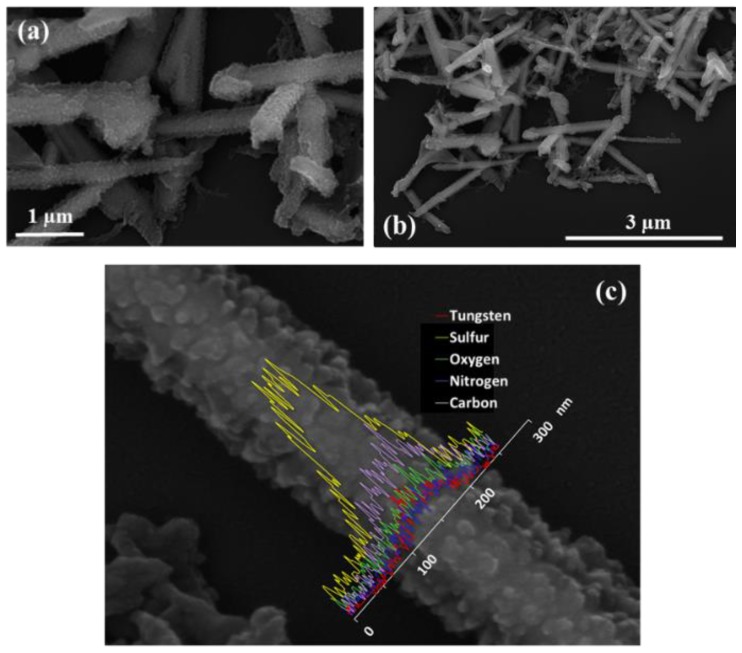
HRSEM images (**a**,**b**) and EDS line scan (**c**) of sample INT-PANI-HA.

HRSEM images of sample INT-PANI-HA (Figure 4a,b) complete the TEM images, presenting a 3-dimensional conformal shell with a spiky texture, coating individual nanotubes. EDS line scan (c) further confirms the core-shell structure of the composite. The PANI coating appears dark, surrounding the lighter WS_2_ nanotube core. The signals for carbon and nitrogen are relatively wide, implying the presence of these elements all around the composite. Signals for tungsten and especially sulfur are a bit narrower with maxima in the lighter core area. 

**Figure 5:**
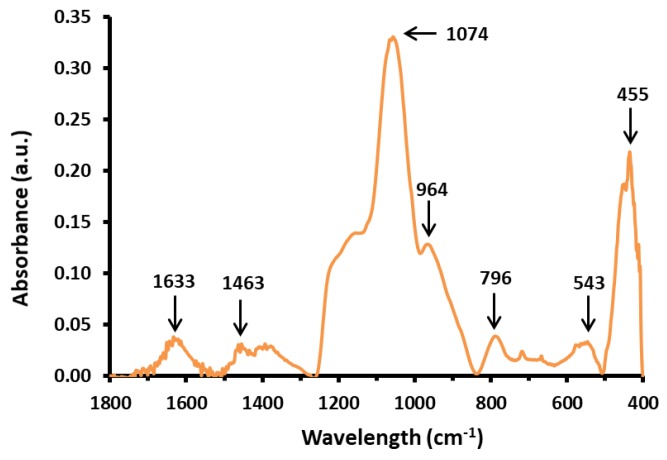
FTIR absorbance spectrum for sample INT-SiO_2_-1P.

The bands in the FTIR absorbance spectra for SiO_2_-coated samples were in good agreement with the literature data about nanocomposites coated with amorphous silica [27,29,32,34,38]. The spectrum of the representative sample INT-SiO_2_-1P is presented in Figure 5. The band at 1074 cm^−1^ is assigned to Si-O-Si asymmetric stretches. The band at 796 cm^−1^ is assigned to Si-O-Si symmetric stretches. The band at 455 cm^−1^ is assigned to O-Si-O bending. The band at 964 cm^−1^ is assigned to Si-OH asymmetric stretches. The weak bands at 1633 cm^−1^ and 1463 cm^−1^ are assigned to O-H bending and wagging.

**Figure 6:**
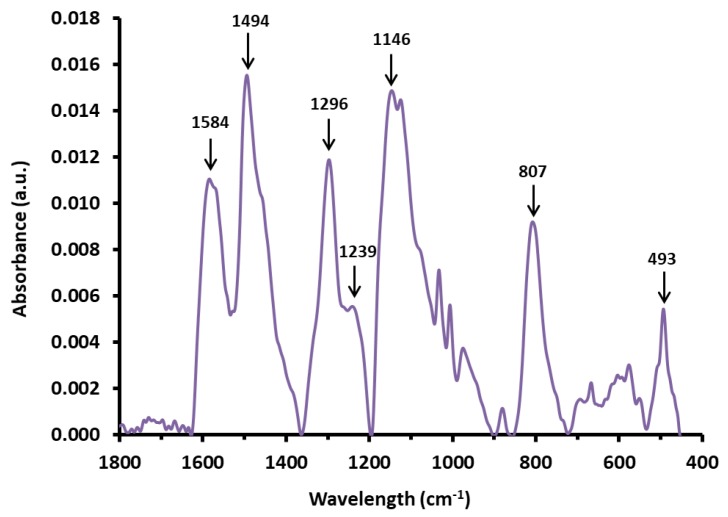
FTIR absorbance spectrum for sample INT-PANI-HA.

FTIR absorbance spectrum of sample INT-PANI-HA (Figure 6) shows typical [71,89,90,91,92,93,94] bands for PANI (this sample is representative as the spectra for all PANI-coated samples looked similar). When aniline rings are connected through their amine groups, the aniline unit within the polymer can be found in a benzenoid (B) form and in a quinonoid (Q) form. The former is a benzene-like ring with 1,4-single bonds to nitrogen atoms and the latter has two carbon-carbon double bonds within the ring and two 1,4-double bonds to the nitrogen atoms. The band at 1584 cm^−1^ is assigned to C=C stretches in the Q ring. The band at 1494 cm^−1^ is assigned to C=C stretches in the B ring. The band at 1296 cm^−1^ is assigned to C-N stretches. The bands at 1146 cm^−1^ and 807 cm^−1^ are assigned to C-H in-plane and out-of-plane bending, respectively. The band at 493 cm^−1^ is assigned to C-N-C bending.

**Figure 7:**
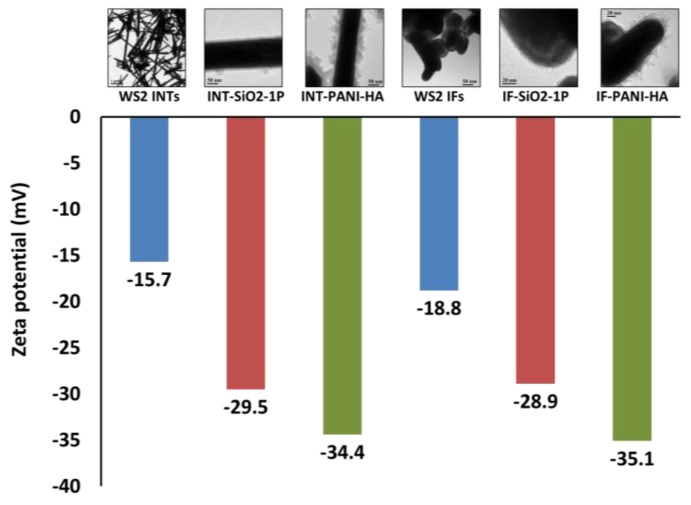
Zeta potentials for aqueous dispersions of bare WS_2_ INTs, WS_2_ IFs and their composites.

To the naked eye, aqueous dispersions of the nanocomposites appeared to be relatively stable overtime, differently aqueous dispersions of bare WS_2_ INTs and IFs, which aggregate after a few minutes. To test this observation, zeta potentials of bare WS_2_ INTs, WS_2_ IFs and their composites were compared. The results in Figure 7 show that indeed, the absolute values of the nanocomposites zeta potentials are significantly higher than those of bare nanostructures; while the former are closer to the aggregation range, the latter indicate dispersions with a moderate stability.

**Figure 8:**
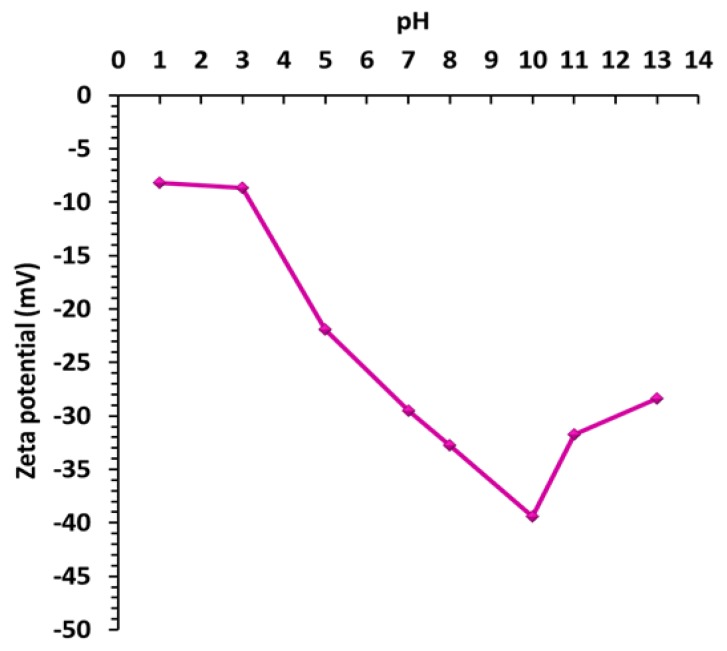
Zeta potential vs. pH for sample INT-SiO_2_-1P.

In addition, zeta potential for an aqueous solution of sample INT-SiO2-1P was measured against pH. The trend in the plot presented in Figure 8 is consistent with the literature [95,96,97]. Zeta potential value decreases with increasing pH, to a minimum of −39.4 mV at pH 10. That is the point of maximum stability (highest absolute value) of the solution. When pH rises above 10, zeta potential starts increasing again. Absolute value is decreasing and so is the stability of the solution. This happens because the ionic strength of the medium increases, compressing the electrical double layer around the particle and allowing attractive van der Waals forces to dominate. Usually, the zeta potential of silica is negative only when pH is above 2–3 but in our case, it is negative through the entire pH range. This phenomenon is typical to silica-coated nanocomposites [98,99].

**Figure 9:**
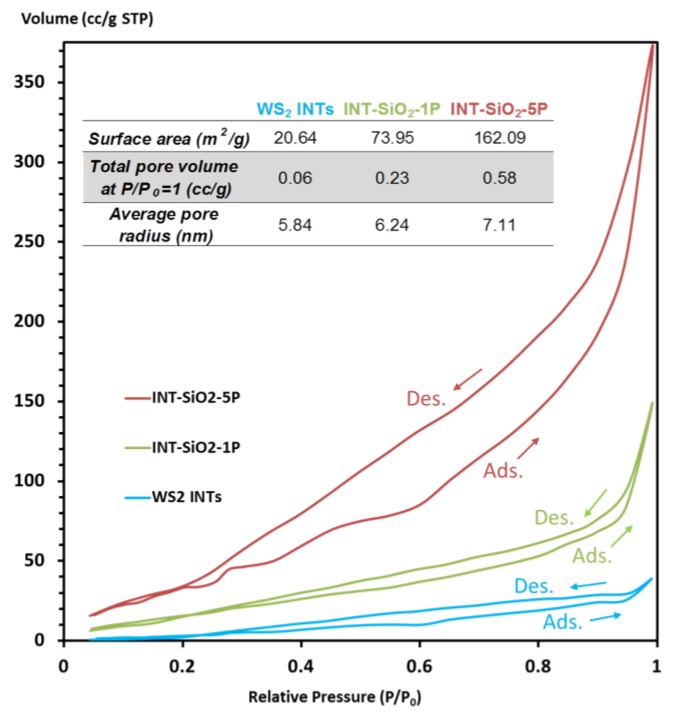
BET N_2_ adsorption-desorption isotherms and data for WS_2_ INTs, INT-SiO_2_-1P and INT-SiO_2_-5P.

Nitrogen adsorption-desorption analysis was run on the silica-coated WS_2_ nanotubes to check porosity of the coating and the data is presented in Figure 9. The average pore radii suggest that the coating is mesoporous. The shape of the isotherms agrees with type IV adsorption-desorption curves (IUPAC classification), which are typical for mesoporous materials. The curves have characteristic hysteresis loops that usually result from capillary condensation within the mesoporous structure [100].

The surface areas of the coated samples are an order of magnitude smaller than that of ordered/crystalline mesoporous silica, which can be hundreds to about a thousand m^2^/g [87,101,102]. This makes sense, as it was previously implied from the TEM images that the coating is amorphous and does not visually resembles ordered mesoporous silica. Nonetheless, there is an evident increase in the surface area and total pore volume when comparing between the coated and non-coated nanotube and between the two coating thicknesses. An interesting option for further modification of the silica coating was using the pseudomorphic transformation method to convert an amorphous silica coating on magnetite nanoparticles to a mesoporous one for DNA plasmid purification [103].

**Figure 10:**
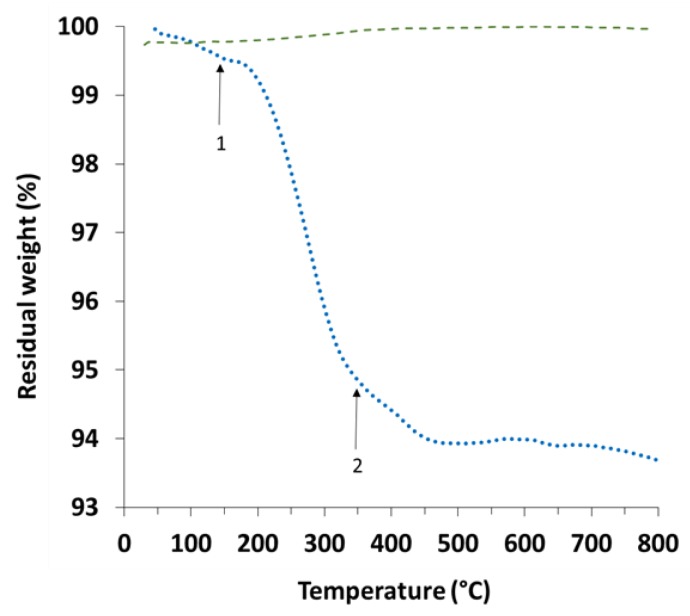
TGA weight loss curve for bare WS_2_ nanotubes (dashed green line) and sample INT-PANI-HA (dotted blue line). The two major weight losses are indicated by arrows.

Figure 10 shows the result of thermogravimetric analysis for bare WS_2_ nanotubes and coated sample INT-PANI-HA. The samples were heated under nitrogen to avoid oxidation of the nanotube. The plot presents an overall weight loss of 6.5%. An initial weight loss of 0.5% occurs under about 150 °C (arrow 1) and is assigned to moisture. In the range of 150 °C to 350 °C there is a weight loss of 5% which may be assigned to residual HCl used in the polymerization process but can also originate from shorter aniline oligomers. Another weight loss starts at 350 °C (arrow 2) and is assigned to the polyaniline backbone. It is interesting to note that although the weight loss ranges are relatively typical to polyaniline [68,73,104], their parts in the weight loss are not. It is expected that the major weight loss will be the one above 350 °C and the case here is different. Because the sample was well-washed before analysis and the pH of the medium was neutral, the contribution of residual HCl should not be significant. It is possible that the PANI shell contains a large number of shorter units, which causes its decomposition to start below 350 °C.

**Figure 11:**
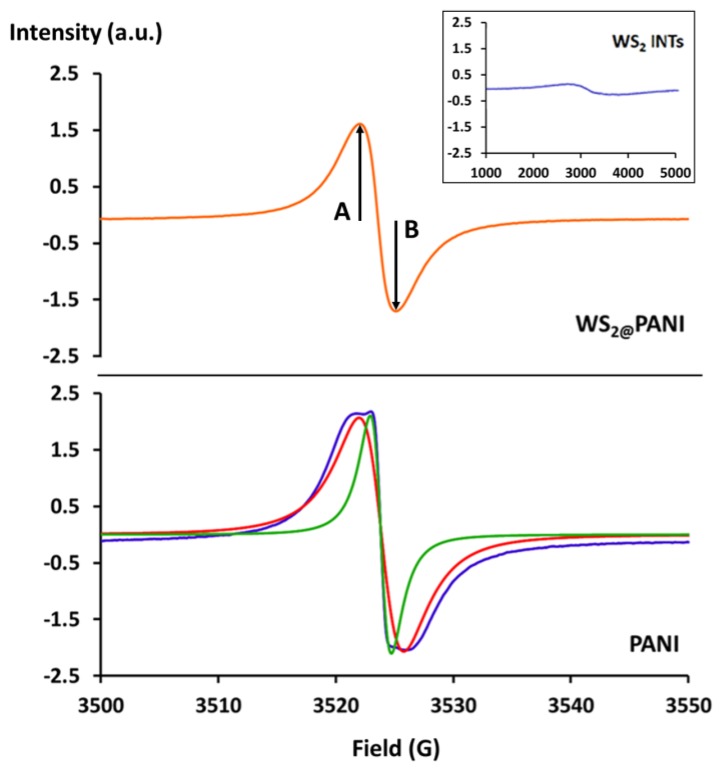
EPR spectra of WS_2_ INTs, PANI and WS_2_@PANI composite. Symmetry parameter A/B is defined in WS_2_@PANI spectrum. Lorentzian peak fittings for PANI spectrum are plotted red and a green.

Figure 11 shows the EPR spectra for bare WS_2_ nanotubes (inset), PANI and WS_2_@PANI composite (sample INT-PANI-SA2 was tested). The PANI sample was prepared in the same manner as the composite sample mentioned above, excluding WS_2_ INTs. EPR signal for bare WS_2_ nanotubes is negligible. An intense EPR peak of free radical without hyperfine structure appears for PANI and WS_2_@PANI samples. This peak is ascribed to the presence of polarons in these samples. The polarons, free electron, in conducting polymers are characterized by electron spin S = ½. Thus, EPR methods are widely used for the study of such systems. Looking carefully at the PANI spectrum, it seems to be composed from two components, a narrow Lorentzian peak (dashed line, *ΔH_pp_* = 1.8 G) and broader one (dotted line, *ΔH_pp_* = 3.8 G). These two components can be related to the bulk polymer polaron spins and to surface polymer polaron spins: the broader component is related to exposure of the polymer surface to oxygen, causing line width increase due to dipole-dipole interaction of the polaron with the paramagnetic oxygen [105]. Another explanation can be that PANI preparation methods still have uncontrolled and incomplete polymerization mechanism. This results in multicomponent product, with different morphologies [106]. It is possible that the PANI consists of different domains having different degree of localization/motion of the polarons domains. Component with decreased localization will have increased spin diffusion and narrow linewidth due to motional exchange narrowing [107,108]. However, WS_2_@PANI EPR is composed from a single component and therefore may reflect a higher degree of structural order of the PANI within the composite, compared to the bulk PANI. The g-values of the EPR signal were 2.0028 and 2.0034, respectively and the *ΔH_pp_* of these signals were 4.4 G and 3 G, respectively.

The g-values are very close to that of the free electron g = 2.0023 or to that of a carbon center radical 2.002–2.003 [109]. They are typical for free radicals of π-system in polyenes and aromatics [110], where the electrons delocalized over the polymeric systems consisting of p_z_ orbitals of carbon and nitrogen atoms in the main chain of polyaniline. In both samples, the observed small line width suggests mobility of the spins and also strong exchange coupling. Similar g-values and *ΔH_pp_* as measured in this work are listed in the literature of PANI [105,108,111,112,113]. The line shape of WS_2_@PANI has a symmetric Lorentzian line with A/B ~1, while the PANI has an asymmetric Lorentzian line shape with A/B ~1.2. This asymmetry may arise from unresolved anisotropy of g-value or, most likely, from the dysonian character of PANI [113]. The dysonian line shape is common, registered in conductive samples such as highly-doped PANI, having a skin depth smaller than the sample thickness [114]. Thus, a non-uniform distribution of the microwave field in the sample causes a dispersive component in the line shape resulting in asymmetry [115]. As Dysonian is accompanied by line shift to a higher magnetic field [108], the decrease in the g-value to 2.0028, compared to 2.0034 in WS_2_@PANI is another indication for the dysonian character of PANI, which is not represented in WS_2_@PANI. We can suggest that WS_2_ decreases the conductivity of PANI with an increase in the skin depth, causing a symmetric peak. One explanation to the possible decrease in the conductivity of WS_2_@PANI can be a decrease in spin density. As PANI coats the WS_2_ surface, there is a decrease in the bulk polymer concentration and as a result, a decrease in the total spin density and in conductivity. This can also explain the decrease in the linewidth of WS_2_@PANI, since the decrease in the spin density will also decrease the dipole interactions of the spins with neighbors.

## 4. Conclusions

Two new core-shell nanocomposites were introduced: WS_2_@SiO_2_ and WS_2_@PANI. Their preparation was based on a preliminary treatment of the WS_2_ nanostructures followed by polymerization of TEOS or aniline on the surface of the WS_2_ INTs or IFs. Structural analysis confirmed the presence and characters of the obtained conformal shells. The silica shell is amorphous and mesoporous. Its thickness and as a result—its surface area, can be adjusted. The stability of the aqueous WS_2_@SiO_2_ dispersion can be increased by pH adjustment.

Polyaniline also formed an amorphous shell around the WS_2_ core. Different acids used in the polymerization process lead to slightly different shell morphologies. EPR of the WS_2_@PANI nanocomposite shows that PANI character within the composite is slightly different from its bulk character. 

This work is merely a first step towards a wider use of TMDC nanostructures. The silica shell is mesoporous and substituted TEOS can be used to produce a hybrid silica shell. PANI is a very versatile conductive polymer which can be prepared under many conditions and with different dopants and it differs in its properties accordingly. In addition, the LbL surface modification done on the core TMDC allows other water-soluble monomers to be tested as shell precursors. We are currently working on testing the nanocomposites described here as enhanced reinforcing agents in polymeric fibers and coatings. For further work, our thoughts include testing WS_2_@SiO_2_ nanocomposites in biomedical applications and WS_2_@PANI for Li-ion batteries.
